# Self-Directed Weight Loss Strategies: Energy Expenditure Due to Physical Activity Is Not Increased to Achieve Intended Weight Loss

**DOI:** 10.3390/nu7075256

**Published:** 2015-07-16

**Authors:** Ulf Elbelt, Tatjana Schuetz, Nina Knoll, Silke Burkert

**Affiliations:** 1 Department of Endocrinology, Diabetes and Nutrition, Charité-Universitätsmedizin Berlin, Charitéplatz 1, 10117 Berlin, Germany; 2 Integrated Research and Treatment Center (IFB) Adiposity Diseases, Core Unit Nutrition and Clinical Phenotyping, Leipzig University Medical Center, Philipp-Rosenthal-Straße 27, 04103 Leipzig, Germany; E-Mail: tatjana.schuetz@medizin.uni-leipzig.de; 3 Health Psychology Department, Freie Universität Berlin, Habelschwerdter Allee 45, 14195 Berlin, Germany; E-Mail: nina.knoll@fu-berlin.de; 4 Institute of Medical Psychology, Charité-Universitätsmedizin Berlin, Luisenstraße 57, 10117 Berlin, Germany; E-Mail: silke.burkert@charite.de

**Keywords:** self-directed weight loss, energy expenditure, physical activity pattern, nutritional habits

## Abstract

Reduced physical activity and almost unlimited availability of food are major contributors to the development of obesity. With the decline of strenuous work, energy expenditure due to spontaneous physical activity has attracted increasing attention. Our aim was to assess changes in energy expenditure, physical activity patterns and nutritional habits in obese subjects aiming at self-directed weight loss. Methods: Energy expenditure and physical activity patterns were measured with a portable armband device. Nutritional habits were assessed with a food frequency questionnaire. Results: Data on weight development, energy expenditure, physical activity patterns and nutritional habits were obtained for 105 patients over a six-month period from an initial cohort of 160 outpatients aiming at weight loss. Mean weight loss was −1.5 ± 7.0 kg (*p =* 0.028). Patients with weight maintenance (*n =* 75), with substantial weight loss (>5% body weight, *n =* 20) and with substantial weight gain (>5% body weight, *n =* 10) did not differ in regard to changes of body weight adjusted energy expenditure components (total energy expenditure: −0.2 kcal/kg/day; non-exercise activity thermogenesis: −0.3 kcal/kg/day; exercise-related activity thermogenesis (EAT): −0.2 kcal/kg/day) or patterns of physical activity (duration of EAT: −2 min/day; steps/day: −156; metabolic equivalent unchanged) measured objectively with a portable armband device. Self-reported consumption frequency of unfavorable food decreased significantly (*p =* 0.019) over the six-month period. Conclusions: An increase in energy expenditure or changes of physical activity patterns (objectively assessed with a portable armband device) are not employed by obese subjects to achieve self-directed weight loss. However, modified nutritional habits could be detected with the use of a food frequency questionnaire.

## 1. Introduction

The prevalence of obesity, which is caused by an imbalance of energy intake and energy expenditure, is increasing dramatically worldwide [1,2]. Numerous chronic diseases are associated with obesity, and of particular importance, obesity is a major risk factor for the development of diseases related to the metabolic syndrome-like type 2 diabetes mellitus, arterial hypertension or dyslipidemia [3,4,5]. Although the specific etiology of some rare forms of obesity (monogenetic obesity, syndromal obesity and endocrine diseases) is known (e.g., [6,7]), the complex and multifaceted reasons for the development of common obesity and its rapid increase in prevalence over the last few decades are still insufficiently understood. Beside the description of genetic factors influencing energy balance and predisposing to the development of common obesity (with each explaining only a few kilograms of weight gain, e.g., [8]) the enormous changes of environmental and behavioral factors with a reduced energy expenditure due to physical (in-)activity [9,10] and an unlimited availability of food are thought to be major contributors [11]. This leads to the question of how the necessary changes of sedentary lifestyle and over-nutrition for the treatment of obesity can be addressed at an individual, as well as at a population level.

Although successful conservative strategies for the treatment of obesity have been described, only a few obese subjects are able to achieve substantial weight loss, and even fewer obese subjects are able to maintain lasting weight loss [12]. In addition, pharmacological interventions have been of limited success [13]. Bariatric surgery procedures seem to be effective for achieving significant weight loss and for reducing obesity-associated morbidity and mortality [14]. However, there is still concern about long-term safety issues and the interventional risk.

A precise assessment of energy expenditure and physical activity in individuals used to depend mainly on self-report questionnaires with the limitation of considerable over-reporting in obese patients [15]. The availability of reliable motion sensors in out-patient settings has certainly helped to improve the assessment of energy expenditure and physical activity and may lead to a better understanding of energy expenditure in individuals at risk of or with obesity [16,17].

Strenuous work or exercise plays a minor role in the energy balance of individuals living in affluent societies. Therefore, spontaneous physical activity or non-exercise activity thermogenesis (NEAT) seems to be a better predictor for the estimation of energy expenditure [18]. Accordingly, Levine *et al.* reported NEAT to be the most variable component of total energy expenditure and hypothesized NEAT to play a crucial role in the pathogenesis of obesity [19]. For lean individuals, it has been shown that the least fat gain occurred in those individuals who increased their NEAT the most during overfeeding [19]. Recently, we have described that body weight-adjusted components of energy expenditure decreased with rising degrees of obesity [20], and in addition, different patterns of physical activity across the week have been described for normal-weight, overweight and obese adults [21]. 

In the medium term, nutritional interventions have been shown to be effective for weight loss [22]. In addition, an observed shift to more healthy dietary patterns over a decade within a large cohort was associated with less weight gain [23]. Usually, food frequency questionnaires to estimate food consumption are used for the complex quantification of energy intake, especially for longitudinal assessment [24]. However, the reliability of these widely-used instruments has some limitations (under-reporting) [24,25]. 

Self-directed weight loss strategies with dieting and exercising are very popular [26], because they are neither very time-consuming, nor expensive. Unfortunately, the effectiveness of these strategies is largely unknown. Information about self-directed weight loss may be deduced from control groups of interventional trials with mean weight loss of −1.4 ± 0.4 kg at six months and −0.8 ± 0.6 kg at 24 months [27].

In this study, we measured (body weight-adjusted components of) energy expenditure (total energy expenditure (TEE), exercise-related activity thermogenesis (EAT)) with a portable armband device and determined body weight-adjusted resting energy expenditure (REE) for the calculation of body weight adjusted NEAT. In addition, we assessed physical activity patterns (duration of EAT, the number of daily steps and mean metabolic equivalent) with the portable armband device. For the estimation of energy intake, we assessed self-reported nutritional habits (frequency of favorable (unfavorable) food consumption, frequency of calorie-free (caloric) drink consumption) with a food frequency questionnaire over a six-month period in patients aiming at self-directed weight loss. To gather naturalistic data, we minimized exclusion criteria and treated the participants of the study in accordance with our routinely-applied therapeutic concepts for patients with intended self-directed weight loss.

Our major aim was to learn more about the strategies obese subjects predominantly utilize for self-directed weight loss in an outpatient setting (increasing energy expenditure, reducing energy intake). We hypothesized that an increase of energy expenditure (especially due to an increase of NEAT with a rise in the number of daily steps and mean metabolic equivalents) would lead to more pronounced weight loss within the six-month study period. Therefore, we analyzed the relationship of changes of energy expenditure, patterns of physical activity and eating behavior with weight development.

## 2. Subjects, Materials and Methods

### 2.1. Subject Recruitment and Exclusion Criteria

A total of 169 outpatients seen consecutively between May 2009 and April 2011 in the Division of Clinical Endocrinology, Charité-Universitätsmedizin Berlin, who came for a diagnostic work-up of obesity and aimed at self-directed weight loss, was asked to participate in an observational study over a 6-month period. Patients lacking sufficient German language skills were not asked to participate in the study. Furthermore, subjects suffering from severe diseases, such as clinically-relevant heart failure, limiting pulmonary disease, severe osteoarthritis or amputation preventing them from performing basic daily physical activities, were excluded. In addition, hypercortisolism as a predefined exclusion criterion was assessed by 24 h urinary free cortisol excretion or in the case of clinical suspicion with an overnight 1 mg dexamethasone suppression test. Nine patients refused to participate (at least two of them most probably due to concealed illiteracy). Finally, 160 patients were included. Patients were followed up for approximately 6 months.

The study was approved by the institutional ethical committee (Protocol Number EA1/129/09), and all subjects gave written informed consent.

### 2.2. Diagnostic Procedures and Treatment

At initial presentation, a complete medical history was taken, and all subjects underwent a physical examination. Body mass was measured to the nearest 0.1 kg and stature to the nearest 0.01 m at study entry, and body mass was assessed again at follow-up. Body mass index (BMI) was calculated as kg/m^2^ and classified according to the World Health Organisation criteria [28]. Thyroid function was assessed, and in the case of insufficient replacement therapy, the dose of *L*-thyroxine was adjusted. In patients without known diabetes mellitus, glucose tolerance was assessed with a 75-g oral glucose load after an overnight fast. In patients with (known) diabetes, therapy was initiated (optimized) with the focus on avoidance of pharmacotherapy promoting further weight gain. If impaired glucose tolerance or insulin resistance was diagnosed, patients were offered to start an off-label therapy with metformin after exclusion of contraindications. All patients received individualized dietary advice by a dietician (with additional qualifications in medical nutrition and diabetes education) focusing on caloric restriction and an increase of physical activity. According to current recommendations of the Deutsche Gesellschaft für Ernährung (German Nutrition Society), patients were advised to take a diet low in fat, low in glycemic index and high in fiber.

### 2.3. Assessment of Energy Expenditure and Physical Activity with the SenseWear™ Armband

Energy expenditure and physical activity patterns were continuously measured for 3 days (preferably 2 weekdays and 1 weekend day) with a portable armband device (SenseWear™ armband, BodyMedia, Inc., Pittsburgh, PA, USA) in an ambulatory setting as described elsewhere [20]. A day was included in the data analysis if the minimum duration of data acquisition was 20 hours 30 minutes a day. The portable armband device utilizes a multi-sensor array including a 2-axis accelerometer, heat flux sensor, galvanic skin response sensor, skin temperature sensor and a near-body ambient temperature sensor. Data were analyzed by using a generalized proprietary algorithm developed by the manufacturer (InnerView™ Professional, Version 6.1, BodyMedia, Inc., Pittsburgh, PA, USA). The number of steps, metabolic equivalents (MET) and the duration of energy expenditure to distinct levels of physical activity graded in MET were calculated. Metabolic equivalents define the energy expenditure related to body weight. One MET is equivalent to 1 kcal/kg body weight/h.

Activity thermogenesis (AT) was calculated according to the equation: AT = total energy expenditure (TEE) − thermic effect of food (TEF) − resting energy expenditure (REE). The TEF was estimated as 10% of TEE and was calculated as TEE × 0.10 [18]. The REE was calculated according to the equation of Müller *et al.* [29] for overweight (1) or obese subjects (2): (1) BMI > 25 to <30: REE (MJ/day) = 0.04507 × weight (kg) + 1.006 × sex − 0.01553 × age (years) + 3.407 or (2) BMI ≥ 30: REE (MJ/day) = 0.05 × weight (kg) + 1.103 × sex − 0.01586 × age (years) + 2.924; for gender: female = 0 and male = 1. Then, REE was converted to kcal/day. Energy expenditure of more than 5 MET was classified as exercise-related activity thermogenesis. Energy expenditure up to 5 MET was classified as NEAT.

The components of energy expenditure were body weight adjusted to allow for inter- (cross-sectional) and intra-individual (longitudinal) comparison.

### 2.4. Assessment of Body Composition

Bioelectrical impedance analysis was performed under standardized conditions (patient supine, arms relaxed at the sides without touching the body and thighs separated) using a BIA 2000-M analyzer (Nutrigard-M™, Data Input, Darmstadt, Germany) at 50 kHz to measure resistance (R) and reactance (Xc). Total body water (TBW) was calculated as 0.69 × height^2^/R + 0.8, and then, fat-free mass (FFM) was calculated as TBW/0.732 [30]. Fat mass was calculated as the difference of body weight and fat-free mass.

### 2.5. Assessment of Nutritional Habits and Energy Intake

Nutritional habits were assessed using a food frequency questionnaire adapted from Renner *et al.* [31]. Patients were asked to report on the frequency of consumption of high- and low-fat meat, sausage, fish, cheese and dairy products on a six-point rating scale (1 = multiple times a day; 2 = once a day; 3 = multiple times a week; 4 = once or twice a week; 5 = once to thrice a month; 6 = rare or never). Furthermore, consumption of butter, margarine, edible oil, gravy, egg, scrambled eggs, grain products, fresh fruit and vegetables, sweets and salty snacks was assessed. Furthermore, drinking habits were measured (frequency of consumption of water or unsweetened tea, milk, sugary beverages, low-calorie beverages or alcoholic drinks) on the above-described scale.

A factor analysis was performed for favorable (low in fat, low glycemic index with the exception of fresh fruit) and unfavorable components (high in saturated fat, high glycemic index) of food. After exclusion of food components with factor loadings of less than 0.5, a food frequency index (mean frequency of consumption of favorable food/mean frequency of consumption of unfavorable food) was calculated.

### 2.6. Grouping of Participants 

Patients were grouped into three categories according to weight development during the 6-month study period. Patients with a weight loss of over 5% of initial body weight were considered to have achieved substantial weight loss [27,32]; patients with weight changes within 5% of initial bodyweight were considered to have maintained weight; and patients with weight gain of more than 5% of initial body weight were considered to have gained weight substantially.

### 2.7. Statistical Analysis

The distribution of the data was determined by using the Kolmogorov–Smirnov test. Results are expressed as means and standard deviations (SD) for parametric data (age, BMI, body fat assessed with bioelectrical impedance analysis, body weight adjusted TEE, body weight adjusted REE, body weight adjusted NEAT, number of steps/day, frequency of favorable (unfavorable) food consumption, food index). Median and quartiles are given for non-parametric data (fat-free mass assessed with bioelectrical impedance analysis, body weight adjusted exercise-related AT, duration of exercise-related AT, mean METs). The chi-square-test (for categorical variables), two-tailed *t*-test (for normally distributed data), Mann-Whitney U-test (for non-parametrical distributed data), one-way ANOVA (for normally distributed data) and Kruskal–Wallis test (for not normally distributed data) were used. For intra-individual comparisons, the two-tailed paired *t*-test (for normally distributed data) and Wilcoxon-test (for not normally distributed data) were used. A factor analysis was used to extract favorable and unfavorable dietary patterns using 10, respectively 12, food groups by using a varimax rotation to enhance the interpretability of the analyzed factors. Pearson’s coefficient of correlation was used to determine the degree of strength for the linear association between normally-distributed variables (change of body weight during the 6-month study period with change of body weight adjusted NEAT and number of steps/day during the 6-month study period). Statistical significance was set at *p* < 0.05. Data were processed and analyzed using IBM SPSS Statistics 20 (IBM Corp, Armonk, NY, USA).

## 3. Results

### 3.1. Subjects

The initial cohort of 160 subjects (34 men, 126 women) had a mean ± SD age of 41 ± 14 years (range, 18 to 74) with a BMI of 43.3 ± 8.3 kg/m^2^ (range, 26.4 to 67.7). Five patients were overweight; 19 patients suffered from class I obesity, 39 from class II obesity and the majority of 97 patients from class III obesity. A total of 111 (69%) patients came to the outpatient clinic for the first time. Seventy-five patients (47%) presented with the intention to undergo bariatric surgery in the long term, whereas 60 patients (38%) preferred conservative weight loss strategies. The remaining patients did not provide specific information on this issue.

For a more comprehensive description of the study cohort, data on preexisting medical condition and medication of the subjects are given in more detail: single cardiovascular risk factors were present in up to 50% of the patients; however, the frequency of cardiovascular complications was low. Comorbidities and medication are summarized in Table 1. Especially drugs that modify energy intake or energy expenditure are described specifically: two (1%) of the subjects took glucocorticoids, and 26 (16%) of the subjects were taking beta-blockers. Thirty-one (19%) patients received a replacement therapy with L-thyroxine, and in another six (4%) patients, this therapy was initiated. Neither clinical history, nor 24 h urinary free cortisol excretions or overnight dexamethasone suppression tests indicated Cushing’s syndrome in any of the patients.

**Table 1:** Preexisting medical condition and medication at baseline (*n =* 160).

Preexisting Medical Condition	*n* (%)	Medication	*n* (%)
Cardiovascular complications	9 (6)	Oral antidiabetic agents *	49 (31)
Diabetes mellitus type 2	34 (21)	Metformin *	47 (29)
Art. hypertension	80 (50)	*Sulphonylurea/glinide*	4 (3)
Dyslipidemia	45 (28)	*DPP-IV-Inhibitor*	3 (2)
Back pain	36 (23)	*Incretin analogue*	3 (2)
Osteoarthritis	55 (34)	Insulin	14 (9)
		*Basal insulin*	9 (6)
		*Prandial insulin*	14 (9)
		Antihypertensive agents	56 (35)
		*Beta blocker*	26 (16)
		*ACE-Inhibitor/sartane*	49 (31)
		*Calcium channel blocker*	19 (12)
		*Diuretic*	36 (23)
		*Others*	5 (3)
		Lipid-lowering agents	22 (14)
		*Statin*	21 (13)
		*Others*	5 (3)
		Glucocorticoids	2 (1)
		L-thyroxine	31 (19)
		Antidepressive agents	18 (11)

* Metformin was also given off-label for prevention of diabetes mellitus type 2; ACE: angiotensin-converting-enzyme; DPP-IV: dipeptidyl peptidase IV.

### 3.2. Body Composition, Energy Expenditure, Pattern of Physical Activity and Nutritional Habits at Baseline

Bioelectrical impedance analysis was performed in 148 patients at baseline. Fat-free mass was 62.6 kg (quartiles: 57.3/71.6). Body fat was 57.4 ± 17.7 kg (range, 23.2 to 121.1), equaling 45.7% ± 7.3% (range, 23.3 to 61.9) of body weight. 

A total of 144 patients wore the portable armband device for a median of three (quartiles: 2/3) days at baseline. The assessment of data with the portable armband device was insufficient in six patients, mainly due to a too short of a wearing time. Therefore, body weight-adjusted data of total energy expenditure and its single components can be described for the remaining 138 patients. Body weight-adjusted TEE estimated by the SenseWear™ armband (SWA) was 27.9 ± 5.7 kcal/kg/day (range, 18.0 to 48.2). REE adjusted to body weight was 17.0 ± 1.1 kcal/kg/day (range, 14.8 to 20.0). Body weight-adjusted NEAT was 6.4 ± 3.4 kcal/kg/day (range, −0.4 to 16.7), and exercise-related AT had a median of 1.1 kcal/kg/day (quartiles: 0.5/2.2) with a median duration of 11 min (quartiles: 4/21) per day. The number of steps/day was 7209 ± 3793 (range, 1251 to 25,211), and patients had a median metabolic equivalent (MET) of 1.1 (quartiles: 1.0/1.3). Complete data for the frequency of consumption of favorable (unfavorable) food was self-reported by 126 (117) patients. Sufficient dietary data needed to calculate the food index were present for 112 patients. For favorable food, the value was 3.8 ± 0.6 (range, 2.6 to 3.7), while unfavorable food was consumed less often, with a value of 4.7 ± 0.6 (range, 3.3 to 6.0), resulting in an index of 0.82 ± 0.16 (range, 0.55 to 1.38). The frequency of consumption of calorie-free (caloric) drinks was self-reported by 132 (135) patients. The median value was 3.5 (quartiles: 3.0/4.0) for calorie-free drinks and 5.0 (quartiles: 4.3/5.3) for caloric drinks.

### 3.3. Attrition Rate

At follow-up after six months, 46 patients (29%) had dropped out of the study. Another nine (6%) patients received cost coverage from their health insurance companies during follow-up and underwent bariatric surgery. As this was an observational study, we did not ask the patients to postpone the surgical procedure due to ethical considerations. The surgical procedure was performed after approximately 3.3 ± 1.6 (range, 1.0 to 6.0) months after study entry. All bariatric patients completed the six-month follow-up, but were excluded from further analysis. Therefore, follow-up data are available for 105 patients. For answering the question of whether completers and drop-outs of our study differed right from the beginning, a comparison of completers and drop-outs at baseline is given in Table 2.

**Table 2:** Subject data, bioelectrical impedance analysis, energy expenditure and pattern of physical activity at baseline according to attrition.

	Completers (*n =* 114)	Drop-outs (*n =* 46)	*p*-Value
Male/female	25/89	9/37	0.740 ^a^
Age (years)	41.5 ± 13.8	38.2 ± 15.3	0.188 ^b^
BMI (kg/m^2^)	43.6 ± 8.3	42.7 ± 8.3	0.547 ^b^
Preexisting medical condition (n (%)):		
*Cardiovascular complications*	5 (4)	4 (9)	0.284 ^a^
*Diabetes mellitus type 2*	23 (20)	11 (24)	0.601 ^a^
*Art. hypertension*	60 (53)	20 (43)	0.295 ^a^
*Dyslipidemia*	34 (30)	11 (24)	0.452 ^a^
*Back pain*	27 (24)	9 (20)	0.572 ^a^
*Osteoarthritis*	43 (38)	12 (26)	0.161 ^a^
Medication (n (%)):			
Oral antidiabetic agents *	33 (29)	16 (35)	0.469 ^a^
*Insulin therapy*	9 (8)	5 (11)	0.547 ^a^
*Antihypertensive agents*	41 (36)	15 (33)	0.687 ^a^
*Lipid-lowering agents*	16 (14)	6 (13)	0.869 ^a^
Bioelectrical impedance analysis:	*(n = 111)*	*(n = 37)*	
*Fat mass (kg)*	57.3 ± 18.4	57.8 ± 15.5	0.875 ^b^
*Fat free mass (kg)*	62.7 (57.4/72.9) ^c^	61.9 (56.6/68.7) ^c^	0.842 ^d^
Energy expenditure:	*(n = 108)*	*(n = 30)*	
*Body weight adjusted TEE (kcal/kg/day)*	27.9 ± 5.5	28.1 ± 6.4	0.875 ^b^
*Body weight adjusted REE (kcal/kg/day)*	16.9 ± 1.2 *(n = 114)*	17.0 ± 1.0 *(n = 46)*	0.962 ^b^
*Body weight adjusted NEAT (kcal/kg/day)*	6.4 ± 3.3	6.2 ± 3.6	0.816 ^b^
*Body weight adjusted EAT (kcal/kg/day)*	1.2 (0.4/2.2) ^c^	1.0 (0.5/2.8) ^c^	0.769 ^d^
*Duration of EAT (min/day)*	12 (4/21) ^c^	9 (5/21) ^c^	0.629 ^d^
*Number of daily steps*	7283 ± 3595	6940 ± 4494	0.664 ^b^
*Mean MET*	1.1 (1.0/1.3) ^c^	1.1 (1.0/1.4) ^c^	0.848 ^d^
First presentation (*n* (%))	82 (72)	29 (63)	0.270 ^a^
Intending bariatric surgery (*n* (%))	61 (54)	14 (30)	0.506 ^a^

* Metformin was also given off-label for the prevention of diabetes mellitus type 2; BMI: body mass index; TEE: total energy expenditure measured with the portable armband device; REE: resting energy expenditure according to [29]; NEAT: non-exercise activity thermogenesis; EAT: exercise-related activity thermogenesis; MET: metabolic equivalent; ^a^ χ^2^-test; ^b^ two-tailed *t*-test; ^c^ median and quartiles are given for non-parametric data; ^d^ Mann–Whitney U-test.

### 3.4. Development of Weight and Body Composition during the Six-Month Study Period and Grouping of Participants According to Weight Development

During the six-month study period, the 105 patients with follow-up data had a statistically-significant weight loss (two-tailed paired *t*-test: *p =* 0.028) of −1.5 ± 7.0 kg (range, −34.4 to 17.1) resulting in a BMI of 42.5 ± 8.2 kg/m^2^ (range, 28.4 to 65.8). Bioelectrical impedance analysis was performed in 95 participants at baseline and after six months. For these patients, the change of fat-free mass (Wilcoxon test: *p =* 0.527) and body fat (two-tailed paired *t*-test: *p =* 0.037) was −0.10 kg (quartiles: −1.5/1.4) and −1.2 ± 5.4 kg (range, −22.9 to 10.4), respectively.

To enable us to study the strategies that obese subjects utilize for self-directed weight loss, we grouped the patients according to the achieved weight loss within the six-month study period. Twenty (19%) subjects achieved substantial weight loss (more than 5% of body weight) with a weight loss of −11.9 ± 7.3 kg (range, −34.4 to −5.0), being equivalent to −9.3% ± 4.7% of body weight (range, −21.8 to −5.0). Seventy-five (71%) subjects maintained their weight (between 5% weight loss and 5% weight gain) with a weight change of 0.0 ± 2.9 kg (range, −6.7 to 5.9), being equivalent to a weight change of 0.0% ± 2.5% of body weight (range, −4.8 to 4.5), and ten (10%) subjects substantially gained weight over the six-month period (weight gain 8.2 ± 3.8 kg (range, 4.8 to 17.1), being equivalent to 6.7% ± 2.0% of body weight (range, 5.1 to 11.5)). For answering the question of whether these subgroups differed at study entry, we analyzed demographic data, energy expenditure and physical activity patterns at baseline. Patients with substantial weight gain were significantly younger (ANOVA: *p =* 0.020). Baseline energy expenditure and the pattern of physical activity did not show significant differences between the subgroups. Further information on these subgroups of patients with substantial weight loss, weight maintenance and substantial weight gain is given in Table 3.

**Table 3:** Subject data, energy expenditure and pattern of physical activity at baseline according to weight development.

	Patients with Weight Loss (*n =* 20)	Patients with Weight Maintenance (*n =* 75)	Patients with Weight Gain (*n =* 10)	*p*-Value
Male/female	5/15	15/60	3/7	0.811 ^a^
Age (years)	44.2 ± 14.0	42.2 ± 13.2	30.1 ± 14.7	0.020 ^b^
BMI (kg/m^2^)	44.4 ± 7.4	43.0 ± 8.3	40.5 ± 9.6	0.469 ^b^
Energy expenditure:	*(n = 17)*	*(n = 73)*	*(n = 9)*	
*Body weight adjusted TEE (kcal/kg/day)*	30.0 ± 5.5	27.5 ± 5.5	30.2 ± 5.5	0.124 ^b^
*Body weight adjusted REE (kcal/kg/day)*	16.8 ± 0.8	17.0 ± 1.1	17.5 ± 1.4	0.287 ^b^
*Body weight adjusted NEAT (kcal/kg/day)*	7.8 ± 3.6	6.1 ± 3.2	7.4 ± 4.0	0.143 ^b^
*Body weight adjusted EAT (kcal/kg/day)*	1.3 (0.9/4.3) ^c^	0.9 (0.4/2.1) ^c^	1.8 (0.9/3.8) ^c^	0.117 ^d^
*Duration of EAT (min/day)*	13 (9/38) ^c^	9 (4/20) ^c^	20 (10/30) ^c^	0.083 ^d^
*Number of daily steps*	7813 ± 2709	7025 ± 3539	9825 ± 4606	0.074 ^b^
*Mean MET*	1.3 (1.1/1.4) ^c^	1.1 (1.0/1.3) ^c^	1.3 (1.2/1.4) ^c^	0.052 ^d^

BMI: body mass index; TEE: total energy expenditure measured with the portable armband device; REE: resting energy expenditure according to [29]; NEAT: non-exercise activity thermogenesis; EAT: exercise-related activity thermogenesis; MET: metabolic equivalent; ^a^ χ^2^-test; ^b^ ANOVA; ^c^ median and quartiles are given for non-parametric data; ^d^ Kruskal–Wallis test.

### 3.5. Development of Energy Expenditure and Pattern of Physical Activity during the Six-Month Study Period

Ninety-one patients wore the portable armband device for a median of three (quartiles: 2/3) days at follow-up. The assessment of data with the portable armband device was insufficient in four patients and for another two patients, sufficient data from baseline did not exist. Therefore, longitudinal data of total energy expenditure and its single components can be given for 85 patients.

Body weight-adjusted TEE showed a non-significant decrease (two-tailed paired *t*-test: *p =* 0.728) with a change of −0.2 ± 5.0 kcal/kg/day (range, −11.7 to 22.2). Body weight-adjusted NEAT decreased statistically non-significantly (two-tailed paired *t*-test: *p =* 0.363) with −0.3 ± 2.9 kcal/kg/day (range, −10.1 to 11.0), and exercise-related AT was reduced statistically non-significantly (Wilcoxon test: *p =* 0.164) by a median of −0.2 kcal/kg/day (quartiles: −0.8/0.6) with a statistically non-significant median reduction in duration (Wilcoxon test: *p =* 0.276) of −2 min (quartiles: −8/6) per day. The number of steps/day decreased statistically non-significantly (two-tailed paired *t*-test: *p =* 0.681) by −156 ± 3485 (range, −8208 to 15133), and metabolic equivalent (MET) did not change (Wilcoxon test: *p =* 0.593) with 0.0 (quartiles: −0.1/0.1). 

**Figure 1:**
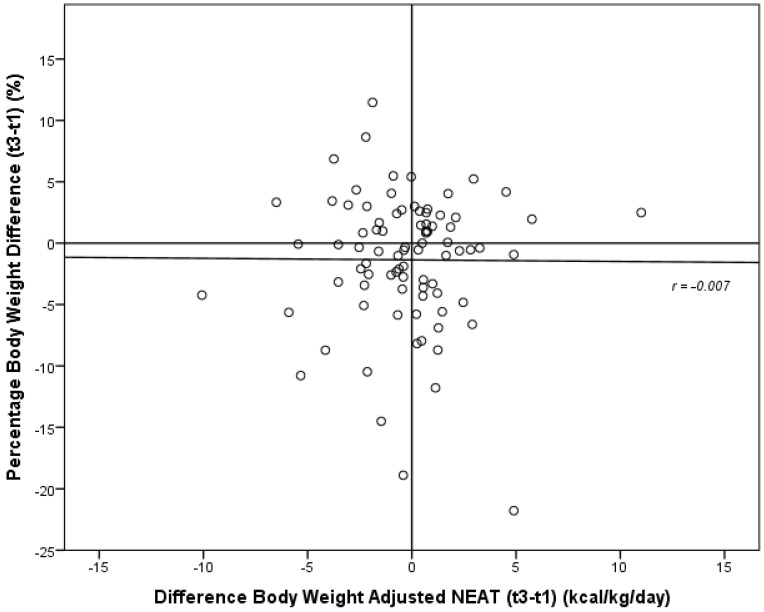
Correlation of the differences of percentage body weight with the difference of body weight-adjusted NEAT between baseline (t1) and six-month follow-up (t3); NEAT: non-exercise activity thermogenesis.

**Figure 2:**
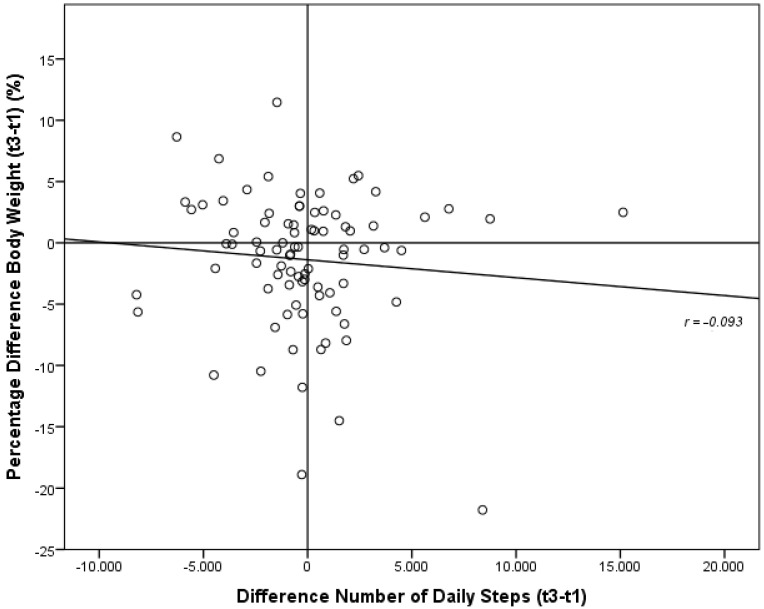
Correlation of differences of the percentage body weight with the difference of number of daily steps between baseline (t1) and six-month follow-up (t3).

For studying the question of whether obese subjects increased energy expenditure for achieving self-directed weight loss, we analyzed the changes of energy expenditure and patterns of physical activity in the subgroups of patients with substantial weight loss, weight maintenance and substantial weight gain. Our results are depicted in Table 4. For further testing our hypothesis that an increase of NEAT with a rise in the number of daily steps would lead to more pronounced weight loss, we correlated percentage body weight loss during the six-month period with the difference of body weight-adjusted NEAT and number of daily steps. These results are shown in Figure 1 and Figure 2. 

**Table 4:** Development of energy expenditure, pattern of physical activity and nutritional habits according to weight development.

	Patients with Weight Loss (*n =* 20)	Patients with Weight Maintenance (*n =* 75)	Patients with Weight Gain (*n =* 10)	*p*-Value
Energy expenditure:	(*n =* 17)	(*n =* 62)	(*n =* 6)	
*Δ body weight adjusted TEE (kcal/kg/day)*	−0.6 ± 4.5	0.2 ± 5.2	−2.6 ± 3.1	0.407
*Δ body weight adjusted REE (kcal/kg/day)*	0.5 ± 0.3	0.0 ± 0.2	−0.4 ± 0.3	0.000
*Δ body weight adjusted NEAT (kcal/kg/day)*	−0.5 ± 2.8	−0.2 ± 3.0	−1.0 ± 2.3	0.762 ^a^
*Δ body weight adjusted EAT (kcal/kg/day)*	−0.1 (−2.0/0.7) ^b^	−0.2 (−0.8/0.6) ^b^	−0.3 (−2.9/0.7) ^b^	0.764 ^c^
*Δ duration of EAT (min/day)*	−1 (−21/9) ^b^	−2 (−7/5) ^b^	−4 (−21/11) ^b^	0.863 ^c^
*Δ number of daily steps*	−179 ± 3333	−15 ± 3553	−1544 ± 3457	0.596 ^a^
*Δ mean MET*	0.0 (−0.1/0.1) ^b^	0.0 (-0.1/0.1) ^b^	−0.1 (−0.2/0.0) ^b^	0.406 ^c^
Δ frequency of favorable food	0.13 ± 0.38 (*n =* 14)	−0.07 ± 0.48 (*n =* 52)	−0.33 ± 0.63 (*n =* 6)	0.131 ^a^
Δ frequency of unfavorable food	0.11 ± 0.55 (*n =* 14)	0.17 ± 0.47 (*n =* 48)	0.05 ± 0.65 (*n =* 6)	0.825 ^a^
Δ food frequency index	−0.01 ± 0.09 (*n =* 12)	−0.05 ± 0.14 (*n =* 44)	−0.07 ± 0.17 (*n =* 6)	0.625 ^a^
Δ calorie-free drinks	0.0 (0.0/0.1) ^b^ (*n =* 17)	0.0 (0.0/1.0) ^b^ (*n =* 61)	0.3 (−0.8/1.5) ^b^ (*n =* 6)	0.989 ^c^
Δ caloric drinks	−0.3 (−0.8/0.2) ^b^ (*n =* 17)	0.0 (−0.3/0.3) ^b^ (*n =* 60)	0.2 (0.0/0.7) ^b^ (*n =* 6)	0.060 ^c^

BMI: body mass index; Δ: difference t3 (6-month follow-up)−t1 (baseline); TEE: total energy expenditure measured with the portable armband device; REE: resting energy expenditure according to [29]; NEAT: non-exercise activity thermogenesis; EAT: exercise-related activity thermogenesis; MET: metabolic equivalent; ^a^ ANOVA; ^b^ median and quartiles are given for non-parametric data; ^c^ Kruskal–Wallis test.

### 3.6. Development of Nutritional Habits during the Six-Month Study Period

At follow-up, complete data for consumption frequency of favorable (unfavorable) food was self-reported by 79 (78) patients. Calculation of food indices was possible for 73 patients. A longitudinal comparison of baseline data with data at the six-month follow-up show a statistically non-significant reduction (two-tailed paired *t*-test: *p =* 0.345) of the food frequency value for favorable food of 0.05 ± 0.48 (*n =* 72). The value of food frequency for unfavorable food significantly increased (two-tailed paired *t*-test: *p =* 0.019) by 0.14 ± 0.50 (*n =* 68), implying a less frequent consumption. Accordingly, the index declined in a statistically-significant manner (two-tailed paired *t*-test: *p =* 0.008) by 0.05 ± 0.13 (*n =* 62), implying a more favorable diet. At follow-up, consumption frequency of calorie-free (caloric) drinks was self-reported by 90 (88) patients. The value for the consumption frequency of calorie-free drinks increased significantly (Wilcoxon test: *p =* 0.019) over the follow-up period (median 0.00, quartiles 0.00/1.00, *n =* 84), implying a less frequent consumption, whereas the value for the frequency of the consumption of caloric drinks remained constant (Wilcoxon test: *p =* 0.994, *n =* 83). For studying the question of whether obese subjects reduce energy intake for achieving self-directed weight loss, we analyzed changes of nutritional habits according to weight development. Data for the subgroups of patients with substantial weight loss, weight maintenance and substantial weight gain are depicted in Table 4.

## 4. Discussion

Our study provides six-month longitudinal data on: (1) weight development; (2) energy expenditure and physical activity patterns objectively measured with a portable armband device; and (3) self-reported nutritional habits assessed with a food frequency questionnaire for 105 out-patients aiming at self-directed weight loss.

### 4.1. Subjects

The high BMI of our cohort of patients might explain the high percentage of patients (47%) intending to undergo bariatric surgery in the long term. Compared to other groups of patients aiming at self-directed weight loss, e.g., users of a commercial web-based weight reduction program [25], the BMI of our cohort is considerably higher (43.3 ± 8.3 *vs.* 31.7 ± 3.2 kg/m^2^). Regarding age, with the majority of patients in their fourth to sixth decade, patients were comparable with the web-users [25] and with patients participating in a conservative multimodal out-patient weight-reduction program (BMI 40.0 kg/m^2^) [33]. Whereas the web-based self-directed weight loss strategy is more frequently used by men (42%), our cohort shows a predominance of women (79%) similar to the patients participating in multimodal out-patient weight-reduction programs, e.g., [25,33]. With 92 patients (58%) suffering from at least one additional component of metabolic syndrome, the patients of our cohort are at high risk for cardiovascular complications.

### 4.2. Attrition Rate

Within the six-month period, 29% of the patients dropped out of the study. The comparison of patients who completed the study and those who dropped out of the study did not show any significant difference between the groups at baseline with respect to gender, age, BMI, preexisting medical condition, medication, body composition, energy expenditure, pattern of physical activity, first presentation at the out-clinic and the intention to undergo bariatric surgery. Self-directed weight loss by using web-based weight-reduction programs shows higher drop-out rates (e.g., 57% [25]) within the first six months compared with drop-out rates reported for multimodal weight-reduction programs (e.g., 24% within the first six months reported by Ahnis *et al.* [33], 39% within 12 months reported by Jebb *et al.* [34]). High premature discontinuation rates seem to be disease-specific and are even reported for both interventional and placebo groups of pharmacological studies with anti-obesity drugs [35]. The majority of our patients presented for follow-up visits in our out-patient clinic. This might be explained with the low threshold for the patients participating in our study (compared to an active use of the web-based program on a regular basis [25], participation in the multimodal weight-reduction program for 2.5 hours twice a week [33] or pharmacological trails [35]) and with the direct physician-patient interaction in our study.

### 4.3. Energy Expenditure, Pattern of Physical Activity at Baseline

The components of body weight-adjusted energy expenditure assessed with the portable armband device at baseline in our group of completers with mainly class III obesity (mean BMI of 43.6 ± 8.3 kg/m^2^) are partly in line with findings we have reported previously [20]. Mean TEE with 27.9 kcal/kg/day was higher than in patients with class III obesity (BMI 48.2 ± 5.3 kg/m^2^, mean TEE 22.1 kcal/kg/day) and those with class II obesity (BMI 37.7 ± 2.0 kg/m^2^, mean TEE 27.3 kcal/kg/day). NEAT with 6.4 kcal/kg/day was comparable to findings of our previous study (obesity class III: 5.7 kcal/kg/day; obesity class II: 9.8 kcal/kg/day). However, energy expenditure due to exercise-related AT with a median of 1.2 kcal/kg/day was higher than the values we reported earlier (obesity class III: median 0.0 kcal/kg/day; obesity class II: median 0.1 kcal/kg/day), and median duration of EAT was considerable higher with 12 minutes/day (obesity class III: 0 minute/day; obesity class II: 1 minute/day). Whereas in another study, mean MET for obese females (mean age 40.9 years) was 1.3 [21], mean MET in completers (mainly women) was lower with 1.1. However, mean reported BMI of the women [21] was 10.2 kg/m^2^ lower (43.6 *vs.* 33.4 kg/m^2^). Drenowatz *et al.* [36] reported a slightly higher body weight-adjusted TEE of 29.5 (33.1) kcal/kg/day for obese women (men) with a lower mean BMI of 32.2 (31.7) kg/m^2^ for women (men). In summary, all of these differences indicate a higher motivation of the participating patients to achieve weight loss by increased physical activity at baseline compared with the obese background population.

### 4.4. Development of Weight, Energy Expenditure, Pattern of Physical Activity and Nutritional Habits

Completers lost statistically significant, 1.5 ± 7.0 kg, amounts of weight during the six-month follow-up period attributed to a significant reduction of body fat assessed with bioelectrical impedance analysis. However, the limitations of this method, e.g., dependency on state of hydration, diurnal variations for resistance measurement, have to be considered [37]. 

Only 19% achieved substantial weight loss of more than 5% of initial body weight, and 10% even substantially gained weight. This achieved self-directed weight loss is clearly lower than weight loss achieved in interventional nutrition trails [38,39], multimodal weight reduction programs [40] and web-based self-directed weight loss [25]. However, mean weight loss for the subjects of the control group enrolled in the Swedish Obese Subjects (SOS) interventional trail after six months who tried to lose weight with professional help (54% out of 1771 controls) was similar [41]. 

Interestingly, and in contrast to our expectations, all different body weight-adjusted components of energy expenditure (TEE, NEAT, exercise-related AT) had decreased in line with reduced duration of exercise-related AT and the number of daily steps at the six-month follow-up. However, all of these changes did not reach statistical significance. Declines of activity during study periods are a frequent finding for groups of patients without specific exercise therapy [42]. Even our patients who achieved substantial weight loss were not able to maintain their baseline level of physical activity. However, the reduction of body weight-adjusted components of energy expenditure (TEE, NEAT, exercise-related AT) and unfavorable changes in physical activity patterns (duration exercise-related AT, number of daily steps, mean MET) were less pronounced compared to patients with weight gain (again without reaching statistical significance). This counterintuitive finding might be explained with the presumably high initial motivation to increase physical activity at baseline and the excitement to monitor physical activity with the portable armband device (reactivity effect), an effect that may not have lasted until follow-up. In line with the existing literature [18,20], energy expenditure due to NEAT is approximately six-fold higher than energy expenditure due to exercise-related AT. The contribution of NEAT to weight gain resistance in humans during a period of overfeeding has been convincingly demonstrated [19]. However, an intentional increase in spontaneous physical activity for self-directed weight loss resulting in increased NEAT as a possible approach for achieving weight loss has, to the best of our knowledge, not been reported so far [43]. Spontaneous physical activity is hypothesized to be an intrinsic heritable trait [44] and to be regulated by brain pathways distinct from those regulating purposeful activity [45].

The self-reported frequency for the consumption of unfavorable food decreased significantly during follow-up; however, the self-reported frequency for the consumption of calorie-free drinks also decreased significantly. Self-reported changes of nutritional habits did not differ between patients with weight loss, weight maintenance and weight gain. These data have to be interpreted with caution [24]. Assessment of self-reported nutritional habits or energy intake may appear idiosyncratic, with higher energy intake in participants with successful weight loss [25] suggesting a more accurate perception of eating habits and energy intake. 

### 4.5. Strengths and Limitations

A strength of our study is the naturalistic design with only a few exclusion criteria, allowing an almost consecutive inclusion of patients attending our out-patient clinic with the intention to achieve self-directed weight loss. Our intervention was very limited in regard to diet and physical activity, with one session of individualized dietary advice focusing on caloric restriction and an increase of physical activity. Thus, results should be representative for this group of patients. Furthermore, our cohort is well characterized with regard to comorbidities and medication. Another strength is the objective measurement of energy expenditure and physical activity patterns with the portable armband device, which limits an over-reporting bias [46,47]. Although there had been concern that the placement of the device on the upper arm might lead to erroneous measurement of ambulation [48], comparisons of several physical activity monitors in (the daily life of) healthy volunteers showed SWA to be the best estimate of energy expenditure [17,49].

However, our study shows several limitations. First, a major limitation is the assessment of nutritional preferences by using a self-reported food frequency questionnaire. Therefore, data might harbor considerable inaccuracy and a bias due to self-reporting [25]. A second limitation might concern an insufficient time period of assessment of physical activity, as Marr and Heady [50] suggest a minimum of seven days to gain a representative assessment of activity thermogenesis, and Scheers *et al.* [21] described the most pronounced differences across the week on Saturdays. We assessed energy expenditure preferably two weekdays and one weekend day to minimize this limitation. With this approach, we tried to reproduce the proportion of weekdays and weekend days. Still, we might have missed assessing the different physical activity patterns on Saturdays and Sundays that have been reported by Scheers *et al.* [21]. However, our approach is in line with a recent study reporting the combination of a weekday and a weekend day being sufficient for the estimation of weekly physical activity behavior [51]. A third limitation is the absence of information about motivational intensity at the beginning and during the follow-up period. Fourth, 47% of patients reported the intention to undergo bariatric surgery in the long term. Therefore, we cannot exclude that this treatment preference affected the self-directed weight-loss strategies of our patients. As bariatric surgery is an effective and recommended therapy for patients with class III obesity or class II obesity with additional cardiovascular risk factors or comorbidities [14,52,53] and patients are increasingly aware of this treatment option, we deliberately chose to include these patients to display a more naturalistic picture. 

## 5. Conclusions

We found that patients aiming at self-directed weight loss do not increase objectively-measured (components of) energy expenditure or show a favorable change of physical activity pattern over a six-month follow-up period. This weight loss strategy is not even employed by patients with successful weight loss of >5% initial body weight. In contrast, self-reported consumption frequency of unfavorable food decreased significantly (*p =* 0.019). Therefore, modifying eating habits with restriction of energy intake may be less challenging for obese subjects than increasing (spontaneous) physical activity intentionally and seems to be the primary strategy of patients to achieve the energy balance that is needed for successful self-directed weight loss.
